# Drivers of bromeliad leaf and floral bract variation across a latitudinal gradient in the Atlantic Forest

**DOI:** 10.1111/jbi.13746

**Published:** 2019-11-24

**Authors:** Beatriz Neves, Camila M. Zanella, Igor M. Kessous, Fernando P. Uribbe, Fabiano Salgueiro, Fernanda Bered, Alexandre Antonelli, Christine D. Bacon, Andrea F. Costa

**Affiliations:** ^1^ Programa de Pós-graduação em Ciências Biológicas (Botânica) Museu Nacional, Universidade Federal do Rio de Janeiro Rio de Janeiro Brazil; ^2^ Gothenburg Global Biodiversity Centre Göteborg Sweden; ^3^ National Institute of Agricultural Botany (NIAB) Cambridge UK; ^4^ Programa de Pós‐graduação em Genética e Biologia Molecular Instituto de Biociências Universidade Federal do Rio Grande do Sul Porto Alegre Brazil; ^5^ Departamento de Botânica Universidade Federal do Estado do Rio de Janeiro Rio de Janeiro Brazil; ^6^ Department of Biological and Environmental Sciences University of Gothenburg Göteborg Sweden; ^7^ Royal Botanic Gardens Richmond UK; ^8^ Departamento de Botânica Museu Nacional Universidade Federal do Rio de Janeiro Rio de Janeiro Brazil

**Keywords:** biogeography, Bromeliaceae, cpDNA, floral bract shape, geometric morphometrics, leaf size, *Vriesea*

## Abstract

**Aim:**

Understanding the complex interaction and relative contributions of factors involved in species and trait diversification is crucial to gain insights into the evolution of Neotropical biodiversity. Here, we investigated the drivers of morphological variation in bromeliads along a latitudinal gradient in a biodiversity hotspot.

**Location:**

Atlantic Forest, Brazil.

**Taxon:**

A species complex in the genus *Vriesea* (Bromeliaceae).

**Methods:**

We measured shape and size variation for 208 floral bracts and 176 leaves in individuals from 14 localities using geometric morphometrics. We compiled data for two chloroplast regions (*matK* and *trnL‐F*) from 89 individuals to assess genetic diversity, population structure and phylogenetic relationships. We tested the influence of climate, altitude and genetic distance on morphological traits using linear statistical models.

**Results:**

Temperature seasonality is a main driver of floral bract shape. Together with precipitation, it also explains changes in leaf size across the latitudinal gradient. Shifts in morphological traits are correlated with genetic structure and partly support the recent taxonomic delimitation proposed for the species complex. The species started to diversify in the Pliocene ca. 5 Mya. We detected a phylogeographical break in species distribution into northern and southern clades between the Bocaina region and the southern portion of the Atlantic Forest.

**Main Conclusions:**

We identify how geography and environmental changes through time shape floral bracts and leaves in similar ways. At highly seasonal sites with lower annual precipitation (in the southern subtropical portion of the Atlantic Forest), leaves are larger and floral bracts are wide‐elliptic, making them better suited for increased water accumulation. In contrast, at less seasonal sites (in the tropical north, where rainfall is more abundant and temperatures are higher), leaves are narrower and floral bracts are lanceolate‐shaped, facilitating water drainage. The biogeographical break we identified suggests a role of tectonic activity and climatic oscillations in promoting species divergence and diversification.

## 1 INTRODUCTION

The high biodiversity found in the Neotropical region is driven by multiple abiotic and biotic factors occurring simultaneously over different spatial and temporal scales (Antonelli et al., 2018; Antonelli & Sanmartín, 2011). Variation through space and environment can promote adaptive responses in species (Morales, De‐la‐Mora, & Piñero, 2018; Nosil, 2012). When phenotypes are correlated with environmental gradients, these responses often arise by selection (local adaptation), phenotypic plasticity, or some combination, and can influence the patterns of gene flow and distribution (Wanderley et al., 2017). Understanding the complex interaction and relative contributions of factors involved in the diversification of species and traits is crucial to gain insights into the evolution of the Neotropical biota.

In plants, the large variation of leaf shape and size reflects physiological demands imposed by environment (Givnish, 1979; Nicotra et al., 2011), and is observed both among and within species (Morello, Sassone, & López, 2018). Shape and size variability may have distinct patterns in distinct organisms, with size being considered more labile with variation mostly influenced by environment (phenotypic plasticity), while shape can be more conserved and variation usually genetically structured (Cardini & Elton, 2009; Chitwood et al., 2014; Maestri et al., 2016).

Vegetative and reproductive traits can undergo different selective pressures. The lability of vegetative traits often prevails over that found for reproductive ones (Chalcoff, Ezcurra, & Aizen, 2008). Reproductive accessory organs such as showy floral bracts have an important role in pollination, and hence reproductive isolation (Bergamo et al., 2019). Floral bracts can have a variety of colours, shapes and scents decisive to reproductive success, besides their function to protect the flowers and therefore are often more conserved than leaves (Pélabon, Armbruster, & Hansen, 2011). Unlike the poorly studied floral bracts, leaves present a well‐known pattern, where small‐sized leaves are often found in dry and hot places, as well as at high latitudes and elevations to avoid transpirational water loss, while large sized leaves are typical of high humidity and warm conditions (Wright et al., 2017).

Within the Neotropical region, the Atlantic Forest is exceptionally diverse (Myers, Mittermeier, Mittermeier, da Fonseca, & Kent, 2000). The past and present environmental dynamics have a role in biome diversification and current species distribution. Both the Pliocene geological processes and subsequent Pleistocene climatic oscillations and the strong gradient across its latitudinal range may lead to genetic and morphological divergence not only within species, but also within complexes of closely related taxa (Alvares, Stape, Sentelhas, de Moraes, & Sparovek, 2013; Turchetto‐Zolet, Pinheiro, Salgueiro, & Palma‐Silva, 2013). *Vriesea* (Bromeliaceae) is one of the most representative genera of Atlantic Forest epiphytes (Ramos et al., 2019), where more than 90% of its 225 species occur (BFG, 2018). Recent diversification of the genus has caused low infrageneric resolution and unclear species boundaries (Costa, Gomes‐da‐Silva, & Wanderley, 2014; Gomes‐da‐Silva & Souza‐Chies, 2017).

One of the many species complexes within *Vriesea* includes *V. incurvata* Gaudich., *V. taritubensis* var. *taritubensis* E. Pereira & I. A. Penna*, V. taritubensis* var. *brevisepala* E. Pereira & I. A. Penna, *V. taritubensis* var. *patens* B. Neves & A. F. Costa and *V. sucrei* L. B. Sm & Read. For decades these taxa have been misidentified with individuals generally assigned to the first described *V. incurvata* (hereafter, ‘*V. incurvata* complex’). The *Vriesea incurvata* complex is widely and continuously distributed across the Atlantic Forest. A recent taxonomic revision using morphological multivariate analysis showed a north–south gradient in the width and length of both leaves and floral bracts and identified five taxa, including a new variety *V. taritubensis* var. *patens* (Neves, Uribbe, Jacques, Zanella, & Costa, 2018). Here, we aim to assess the drivers of morphological trait variation in the *V. incurvata* complex along a latitudinal gradient in the Atlantic Forest. We build upon previous work (Neves et al., 2018) using geometric morphometrics and the estimation of genetic structure and diversity. We evaluate shape and size of floral bracts and leaves of taxa in the complex. We use climatic, altitudinal and plastid genetic data to address the roles of both historical gene flow and environment in shaping morphological traits and species limits.

We test for trait variation in accordance with two hypotheses: (a) if shape is less labile than size (Cardini & Elton, 2009; Maestri et al., 2016) and is diagnostic for taxa (Neves et al., 2018), then we expect genetic structure to be a main predictor; (b) if vegetative characters are more labile than reproductive ones (Pélabon et al., 2011), then we expect variation in leaf traits to be better predicted by environmental shifts, while variation in floral bracts should be better predicted by genetic divergence.

We interpret our results in the light of the traditional taxonomic delimitation of the complex and the historical biogeographical patterns that can predict current distribution of taxa. By identifying the factors driving trait variation along the Atlantic Forest, we identify the importance of seasonality and rainfall regime as predictors of plant morphology and the influence of abiotic processes on the Atlantic Forest, including the effect of Pleistocene climatic oscillations on current species distribution and genetic diversity.

## 2 MATERIALS AND METHODS

### 2.1 Species and locality description

**Figure 1:**
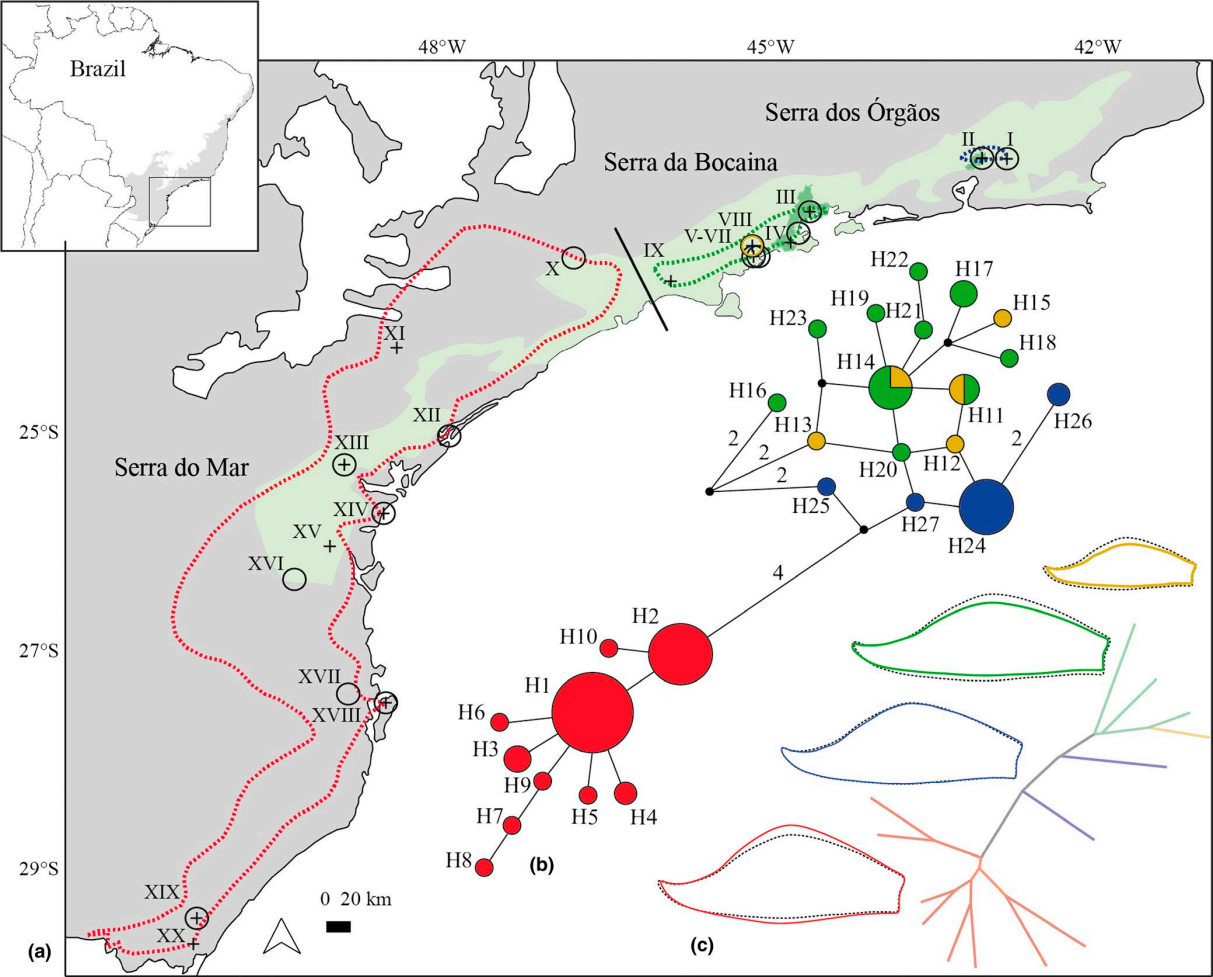
(a) Distribution of taxa based on herbarium records: *Vriesea taritubensis* var. *brevisepala* in blue, *V. taritubensis* var. *taritubensis* in green, *V. taritubensis* var. *patens* in yellow and *V. incurvata* in red. Sampling localities marked with a circle for morphological data and a cross for genetic data (see codes in Table S1, Appendix S2). The Atlantic Forest (grey), Serra do Mar (light green) and Serra dos Órgãos and Serra da Bocaina (dark green) are shown. The black line shows a major break in species distribution between the Serra da Bocaina region and southern Atlantic Forest. Map of Brazil with the Atlantic Forest area in upper left. (b) A median‐joining network shows two main haplogroups, only *V. taritubensis* var. *taritubensis* and *V. taritubensis* var. *patens* shared haplotypes; each circle represents one haplotype, with its diameter proportional to its total frequency; more than one mutational step are indicated by numbers. (c) The Neighbour‐joining tree shows the distance in bract shape among localities. The consensus shapes for each taxon (coloured line) and for the whole sampling (black dotted line) display changes from lanceolate to wide‐elliptic [Colour figure can be viewed at http://wileyonlinelibrary.com]

Taxa within the *V. incurvata* complex are abundant and widely distributed throughout the south‐eastern and southern part of the Atlantic Forest. *Vriesea incurvata* is the most widespread species and *V. taritubensis*, with its three varieties, is restricted to the northern portion of the distribution range. The large populations of *V. taritubensis* var. *brevisepala* are mostly concentrated in the Serra dos Órgãos region, Rio de Janeiro state and *V. taritubensis* var. *taritubensis* and *V. taritubensis* var. *patens* are restricted to the Serra da Bocaina region, Rio de Janeiro and São Paulo states, both mountains are part of the Serra do Mar mountain chain (see Appendix S2: Table S1, Figure 1a). All individuals are epiphytes generally found near rivers and waterfalls, in altitudes varying from 0 to 1,000 m a.s.l., from Rio de Janeiro (22°31′ S) to Rio Grande do Sul (29°44′ S) states (Neves et al., 2018), Brazil. The most notable attribute of these species is their showy red floral bracts covering the inflorescences, filled with a transparent and odourless mucilage. Leaves are highly polymorphic within and among taxa (Neves et al., 2018). Their spiral disposition forms a rosette, and together with leaf sheath size and shape these factors are important for determining the capacity of leaves for water and nutrient storage (Benzing, 2000). Besides the species *V. incurvata* and *V. taritubensis* (with three varieties), Neves et al. (2018) distinguished another species, *V. sucrei* L.B.Sm. & Read, the most divergent in the complex. Its inclusion was needed to determine the position of another binomial involved in the taxonomic revision but we did not include *V. sucrei* in the present work as it is clearly morphologically distinct.

The Atlantic Forest presents high latitudinal (ca. 27°S) and altitudinal (0–2,892 m a.s.l.) variation. Along the study area, the climate ranges from tropical with dry winter at Serra dos Órgãos to humid subtropical with hot summer or temperate summer towards the south, with no distinct dry season. Warmer temperatures are registered in the southeast ranges of both varieties of *V. taritubensis*, and a cooler and marked seasonal climate is found further south where *V. incurvata* occurs (Alvares et al., 2013; Ribeiro, Metzger, Martensen, Ponzoni, & Hirota, 2009). Precipitation ranges from 4,000 mm/year in the coast to 1,000 mm/year in inland areas. Higher precipitation levels are registered especially for Serra da Bocaina region (23°S), explained by the orographic effect caused by the mountains, within the range of *V. taritubensis* var. *taritubensis* and *V. taritubensis* var. *patens* (Table S1, Appendix S2).

### 2.2 Geometric morphometrics

To explore shape and size variation we sampled and georeferenced individuals from 14 localities, 208 individuals for floral bract and 176 individuals for leaf datasets (Figure 1; Table S1, Appendix S2). We selected localities based on a previous study of herbarium collections (Neves et al., 2018), considering accessibility and availability of individuals. We sampled localities spread out along the whole geographical distribution of species, including the type localities of *V. incurvata* and *V. taritubensis*. We randomly sampled 8–19 individuals per locality.

We scanned one floral bract from the middle of the inflorescence and one leaf from the middle of the rosette of each specimen. We then characterized shape by a set of seven landmarks for bracts profile (bracts in side view) and nine for leaves. We discriminate landmarks into anatomical, when located at homologous points with biological meaning; mathematical, when located at extreme points with some geometrical property; and pseudo‐landmarks, when located between other landmarks (Dryden & Mardia, 2016). For bract profile, we placed three anatomical landmarks (1, 2, 5) at the base and apex; two mathematical landmarks (6, 7) and two pseudo‐landmarks (3, 4), at the widest point of the margin and its dorsal opposite and at the midpoint of margin contraction and its dorsal opposite. Such landmarks at the greatest and least width of bract margins reflect greater or lesser cell activity, and their dorsal opposites are needed to minimize the distance among base and tip describing the protuberance they can present (Figure S1a, Appendix S2). The same for leaf, we placed three anatomical landmarks (1, 2, 6) at the sheath base and apex; four mathematical landmarks (3, 4, 8, 9) at sheath widest points and at the transition sheath/blade; and two pseudo‐landmarks (5, 7) at the level 3/4 of blade (Figure S1b, Appendix S2). We used these set of landmarks to better describe the shape of structures without making use of numerous equidistant pseudo‐landmarks along the contour itself (Bensmihen et al., 2008) or the Elliptical Fourier Descriptors (Chitwood & Otoni, 2017), alternatives when homologous points are lacking. To be consistent, a single person (B. Neves) manually digitized the landmarks for each image using TpsDig 2 (Rohlf, 2010). We imported the file containing raw data to MorphoJ 1.06b (Klingenberg, 2011). We then extracted shape information performing a Generalized Procrustes Analysis which removes size, rotation and translation effects (Rohlf & Slice, 1990).

We examined potential bias due to variation within a single individual by testing multiple leaves spirally arranged at the middle of the rosette through a Multivariate Analysis of Variance and Principal Component Analysis (PCA) plot. We also tested for digitization error using Procrustes ANOVA in two sets of data (original and replicated measures of the same leaf) following Klingenberg and McIntrye (1998). We analysed leaves and floral bracts separately, as well as shape and size. We used the centroid size (the square root of the sum of all squared distances from each landmark to the centroid) as a measure of size. We tested allometry by performing a regression analysis between shape and size of each bract and leaf. Because size predicted 47% of leaf shape variation (and 2% for bract), we ran further analyses with the size‐corrected shape matrix resulting from the residuals of the regression. We examined shape and size data performing Procrustes ANOVA, PCA and Canonical Variate Analysis (CVA) (Campbell & Atchley, 1981; Neff & Marcus, 1980), testing for differences among localities and taxa. Due to the marked differentiation in bract shape reflecting the studied taxa, we made a Neighbour‐joining (Saitou & Nei, 1987) tree using Mahalanobis distances, appropriate for morphometric data (Mahalanobis, 1936) and reconstructed the consensus shape for each taxon. All analyses were performed in MorphoJ except for ANOVA and Neighbour‐joining using package ‘vegan*’* (Oksanen et al., 2017) in r 3.5.0 (R Core Team, 2018).

### 2.3 Genetic analyses

To assess genetic diversity and population structure, and to infer the relationships among individuals, we concatenated sequences from two chloroplast regions: *trnL‐trnF* intron and intergenic spacer and *matK* gene for 89 individuals in 15 localities. Among the 15 localities, we have morphological data for nine of them. We collected genetic data independently from the morphological data, individuals were randomly sampled according to availability in the field. We used plastidial data (*trnL‐trnF* and *matK*) from *V. incurvata* sequenced by Zanella, Palma‐Silva, Goetze, and Bered (2016) and from 40 new samples of *V. taritubensis* (Table S1, Appendix S2). Data were deposited in GenBank with accession numbers MN593031 ‐ MN593118*.* For details about DNA processing and PCR conditions see Zanella et al. (2016). We performed multiple sequence alignment in MUSCLE (Edgar, 2004), implemented in MEGA 7.0 (Kumar, Stecher, & Tamura, 2016). Mononucleotide repeat length variations were excluded and indels of more than 1 bp were coded as single mutational event. Using concatenated sequences, we estimated haplotype (*h*) and nucleotide (*π*) diversity (Nei, 1987) for each taxon using ARLEQUIN 3.5.2.2 (Excoffier & Lischer, 2010). The software DnaSP 5.10.01 (Librado & Rozas, 2009) was used to identify haplotypes and calculate distance measures (pairwise *F*_ST_; Wright, 1951) for each pair of locality. The pairwise *F*_ST_ estimatives among localities were displayed in a heat‐map plot using the r package ‘ggfortify*’* (Tang, Horikoshi, & Li, 2016). We inferred the genealogical relationships among haplotypes using the median‐joining method (Bandelt, Forster, & Röhl, 1999) with Network 5 (available at http://www.fluxus-engineering.com). We then performed an analysis of molecular variance to assess the partition of genetic diversity among taxa and localities, using ARLEQUIN with 10,000 permutations.

We ran a Bayesian analysis in BEAST 2.5.2 (Bouckaert et al., 2014) to estimate the temporal divergence of species and varieties using the CIPRES Science Gateway 3.3 (Miller, Pfeiffer, & Schwartz, 2010). *Alcantarea imperialis* (Carrière) Harms, *Goudaea chrysostachys* (É. Morren) W. Till & Barfuss and *Goudaea ospinae* (H. Luther) W. Till & Barfuss were included as out‐group. The HKY nucleotide substitution model was used for each marker separately in MEGA. The uncorrelated lognormal relaxed clock and the birth‐death model (Gernhard, 2008) was used to date the tree. Markov chains were run for 150,000,000 steps, sampling every 15,000 steps, totalling 10,000 trees. We used secondary calibration to date the tree based on Givnish et al. (2014) and Kessous et al. (2019): (a) *Goudaea* + *Alcantarea* + *Vriesea s.s.* node (10 ± 2 Mya), (b) *Alcantarea* + *Vriesea s.s.* clade (8 ± 2 Mya) as enforced monophyletic group and (c) *Vriesea s.s.* (5 ± 1.5 Mya). We evaluated that effective sample sizes for all main parameters were above 200 in Tracer 1.6 (Rambaut, Suchard, Xie, & Drummond, 2014). After discarding 10% of the trees as burn‐in we used TreeAnnotator 1.8.4 (Bouckaert et al., 2014) to generate the maximum clade credibility tree. The software FigTree 1.4.2 (Rambaut, 2014) was used to draw the tree.

### 2.4 Climate and topography

To identify the most important environmental variables in explaining trait variation, we extracted a total of 19 bioclimatic variables from 20 localities (Table S1, Appendix S2). Data were obtained from the Worldclim database (Hijmans, Cameron, Parra, Jones, & Jarvis, 2005) with a spatial resolution of 30 arc‐seconds. In addition, data on altitude were extracted from GPS coordinates in the field. We first performed a PCA with the correlation matrix of all environmental variables to avoid colinearity and overfitting the full model. We used the packages ‘raster*’* (Hijmans et al., 2015) to extract climate variables and ‘stats*’* to perform a PCA in r. The results of the PCA are described in Appendix S1. Because the first three principal components accounted for 96% of variance and were highly correlated with temperature seasonality (BIO4), annual precipitation (BIO12) and altitude (Table S2, Appendix S2), we proceeded the further analyses using these three variables.

To investigate the correlation of the genetic structure, bioclimatic variables and altitude with morphometric traits variation, we ran multiple linear models and to identify the best model in explaining traits variation, we performed model selection based on AIC (Burnham and Anderson 2002). We ran analyses using the R package ‘stats*’* and *‘*MuMIn*’*. We included data only for localities where both genetic and morphological data were collected (9 of the 20 localities Table S1, Appendix S2). We used floral bract shape, floral bract size, leaf shape and leaf size, separately, as response variables. The models were fit with genetic distance across localities, temperature seasonality, annual precipitation and altitude as explanatory factors (response variable ~ pco1gen + pco2gen + temp season + annual prec + alt). To describe shape, we used the regression scores from the size versus shape interaction exported from MorphoJ that captured size‐free information, then we performed a PCA with those regression scores and summarized shape in one value using the first PC (which explained 61% of bract shape and 71% of leaf shape variance) as the descriptor. For size, we used the log‐transformed centroid to control for large variation of size in the sampling (Klingenberg, 2016). To describe genetic distance, we first calculated the pairwise *F*_ST_ between localities using DnaSP. *F*_ST_ is frequently used as a measure of distance for depicting genetic variation (Wright, 1951). Then we performed a Principal Coordinate Analysis (PCoA) on the pairwise *F*_ST_ matrix using the r package ‘ape*’* (Paradis, Claude, & Strimmer, 2004) and selected the first two PCo following the broken stick criterion (Jackson, 1993; Figure S2a, Appendix S2). We used the scores of PCo 1 and PCo 2 as the descriptors for genetics. The PCo1 separates *V. taritubensis* from *V. incurvata*. The PCo2 separates all taxa, both the two species and *V.taritubensis* varieties (Figure S2a, Appendix S2). For climate we used the raw values of the least correlated variables showing the highest loadings in the first three axes of PCA (Table S2, Appendix S2). All explanatory variables were standardized to zero for the mean and unit variance. Additionally, we tested for spatial autocorrelation in size and shape of bract and leaf with Moran's correlograms using the r package ‘ncf*’*. Because significant values were found for bract shape, we corrected for spatial autocorrelation effects by performing Principal Coordinates of Neighbourhood Matrices (PCNM; Borcard & Legendre, 2002) with the r package ‘vegan*’*. The Euclidean distance matrix of the geographical coordinates from the localities was converted into a truncated distance matrix, focusing on neighbouring sites which is then subject to a PCoA. The PCNM axes were used as explanatory variables for bract shape and the one that showed significant explanatory power (PCNM 1) was then incorporated to the model.

## 3 RESULTS

### 3.1 Trait variation

#### 3.1.1 Floral bract

**Figure 2:**
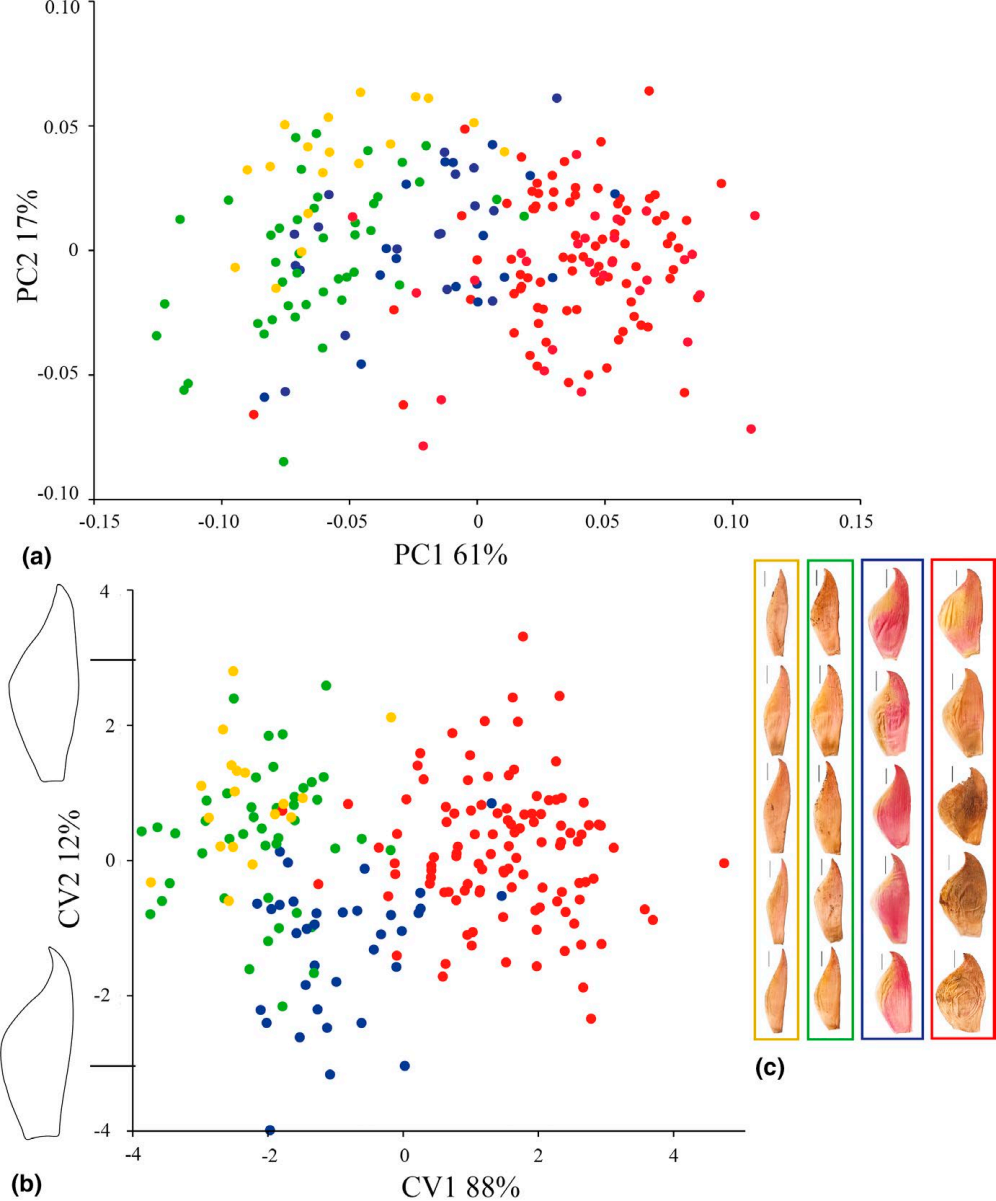
(a) Principal Component Analysis and (b) Canonical Variate Analysis of floral bract landmarks showing taxa divergence. Shape changes in first axes of both analyses are congruent with the consensus taxa shapes in Figure 1. Shape changes in CVA axis 2 relate to a marked contraction in bract apex and an enlargement of the base, found mostly in the blue group. Percentage of total variance for each axis is indicated in *Vriesea taritubensis* var. *patens* in yellow, *Vriesea taritubensis* var. *taritubensis* in green, *V. taritubensis* var. *brevisepala* in blue and *V. incurvata* in red. (c) A subset of bract profiles for each taxon showing lanceolate, elliptic and wide‐elliptic shape, from the left to the right. [Colour figure can be viewed at http://wileyonlinelibrary.com]

The shape of floral bract significantly discriminated three of the four taxa as currently defined morphologically (*df* = 3, *F* = 140.8, *p* < 2e‐16). We identified a shift from lanceolate to wide‐elliptic along the Atlantic Forest latitudinal gradient. In the north, individuals of *V. taritubensis* var. *brevisepala* present elliptic bracts (localities I and II); further south individuals of *V. taritubensis* var. *taritubensis* and *V. taritubensis* var. *patens* have lanceolate bracts (central localities III‐VIII); and individuals of *V. incurvata*, spread throughout the southern portion of Atlantic Forest, with wide‐elliptic bracts (localities IX‐XX; Figure 1). Based on the Neighbour‐joining tree of shape, *V. incurvata* and *V. taritubensis* var. *taritubensis* plus *V. taritubensis* var. *patens* were the most morphologically distant groups (Figure 1c). The PCA and CVA showed clear divergence in floral bract shape, mainly between *V. incurvata* and *V. taritubensis* var. *taritubensis* plus *V. taritubensis* var. *patens*, the last two greatly overlapped in morphological space (Figure 2). The first and second PCs accounted for 61 and 17% of total variation and first and second CVs accounted for 88 and 12% of total variation, respectively. The floral bract shape changes in the first axes of both PCA and CVA analyses are congruent with the consensus taxa shapes of Figures 1c and 2c. Shape changes in CV2 relate to a marked contraction of bract apex and an enlargement of the base, found mostly in individuals of *V. taritubensis* var. *brevisepala* (Figure 2b).

**Figure 3:**
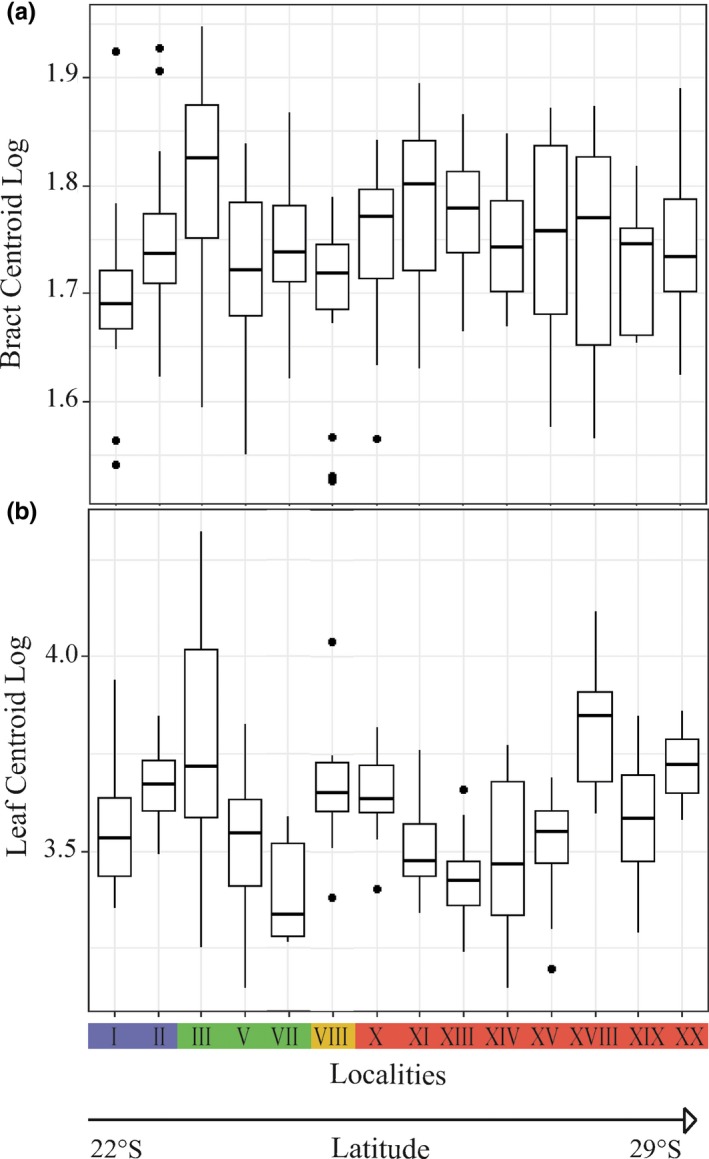
Boxplots of centroid size variation of (a) bracts and (b) leaves in a latitudinal gradient across 14 localities. *Vriesea taritubensis* var. *brevisepala* in blue, *V. taritubensis* var. *taritubensis* in green, *Vriesea taritubensis* var. *patens* in yellow and *V. incurvata* in red. For localities number see Table S1 in Appendix S2 [Colour figure can be viewed at http://wileyonlinelibrary.com]

There was significant difference in bract size among taxa (*df* = 3, *F* = 3.48, *p* = .0168) and among localities (*df* = 13, *F* = 2.03, *p* = .0199). Differences in size separated *V. taritubensis* var. *patens*, with the smallest bracts (locality VIII), of *V. incurvata*, with the largest ones. The locality III presented the longest bracts and localities X‐XX had the largest medium centroid size due to the wider bracts (Figure 3a).

#### 3.1.2 Leaf

**Figure 4:**
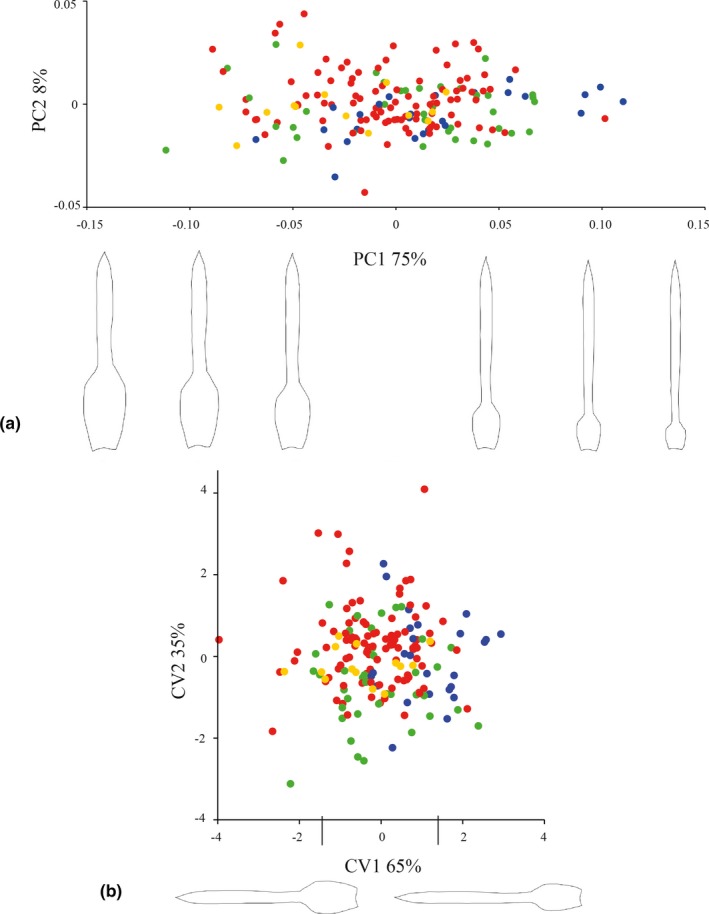
(a) Principal Component Analysis and (b) Canonical Variate Analysis computed on leaf landmarks. Variation of shape from narrow‐obovate to linear is shown along the first PC. The first CV relate to a change of leaf sheaths from large‐obovate to elliptic which separate blue and red groups. Percentage of total variance for each axis is indicated. *Vriesea taritubensis* var. *taritubensis* in green, *Vriesea taritubensis* var. *patens* in yellow, *V. taritubensis* var. *brevisepala* in blue and *V. incurvata* in red [Colour figure can be viewed at http://wileyonlinelibrary.com]

A significant difference in leaf shape between taxa (*df* = 3, *F* = 4.919, *p* = .00265) and localities (*df* = 13, *F* = 2.87, *p* = .000945) was indicated. The PCA showed shape changes from narrow‐obovate to linear along the first PC, which accounted for 75% of the total variance (Figure 4). Despite the overlap due to the high variation within and between localities, we detected a gradual change pointing to the separation of *V. taritubensis* var. *brevisepala* that represents an enlargement of leaf sheaths, from large‐obovate to elliptic (see first CV of CVA in Figure 4b).

Leaf size did not discriminate taxa (*df* = 3, *F* = 0.603, *p* = .614), but differed among localities (*df* = 13, *F* = 7.24, *p* < .0001). The strongest differentiation was among the individuals of *V. taritubensis* var. *taritubensis* (with the largest leaves found in locality III and the smallest in locality VII) and within *V. incurvata* (locality XIII presents the smallest, and localities XVIII and XX the largest leaves) (Figure 3b). Changes in size translated into long, narrow leaves in *V. taritubensis* var. *taritubensis* and *V. taritubensis* var. *patens* and short and wide leaves in *V. incurvata*.

### 3.2 Genetic structure and diversity

We identified 24 polymorphic sites (8 transitions, 4 transversions and 12 indels) resulting in 27 haplotypes from the cpDNA dataset (Table S1, Appendix S2). *Vriesea incurvata*, the most widespread taxon, presented the highest number of haplotypes, followed by *Vriesea taritubensis* var. *taritubensis*. The four taxa showed high haplotype and nucleotide diversity (Table 1). The main source of genetic variation was found among taxa (79%), followed by 17% within localities and 2% among localities within taxa (Table S3, Appendix S2). We found high genetic differentiation among taxa through pairwise *F*_ST_ estimates, excepted between *V. taritubensis* var. *taritubensis* and *V. taritubensis* var. *patens* that showed low genetic differentiation (Table 2). On the other hand, low genetic distance across localities within each taxon was observed (Figure S2, Appendix S2). Two main haplogroups were found: one corresponding to *V. incurvata*, another to *V. taritubensis*. A subgroup can also be identified dividing *V. taritubensis* var. *brevisepala* from *V. taritubensis* var. *taritubensis* and *V. taritubensis* var. *patens* (Figure 1b). Haplotype H2 from *V. incurvata* is separated from H25 and H27 belonging to *V. taritubensis* var. *brevisepala* by five mutational steps and one median vector. A slight divergence was found among the varieties of *V. taritubensis* (Figure 1b). *V. taritubensis* var. *taritubensis* and *V. taritubensis* var. *patens* shared two haplotypes (H11 and H14, Figure 1b).

**Table 1:** Number of specimens (N), number of haplotypes (NH), haplotype diversity (*h*) and nucleotide diversity (*π*) for each taxon and total sampling

Taxa	*N*	NH	*h* (*SD*)	*π* (*SD*)
*V. incurvata*	49	10	0.6922 (0.0499)	0.000404 (0.000299)
*V. taritubensis* var. *taritubensis*	18	9	0.8627 (0.0609)	0.000672 (0.000453)
*V. taritubensis* var. *brevisepala*	16	4	0.4417 (0.1446)	0.000350 (0.000282)
*V. taritubensis* var. *patens*	6	6	1.0000 (0.0962)	0.001299 (0.000886)
Total	89	27	0.8828 (0.0207)	0.001802 (0.000982)

**Table 2:** Pairwise genetic divergence (*F*_ST_ values) for *Vriesea incurvata* complex species based on plastid sequence data (*trnL‐trnF* + *matK*)

	*V. incurvata*	*V. taritubensis* var. *taritubensis*	*V. taritubensis* var. *brevisepala*	*V. taritubensis* var. *patens*
*V. incurvata*				
*V. taritubensis* var. *taritubensis*	0.85024			
*V. taritubensis* var. *brevisepala*	0.85073	0.62717		
*V. taritubensis* var. *patens*	0.82459	0.09474	0.48859	

Note

The *p‐values* determined by permutation test with 10,000 permutations are all significant.

**Figure 5:**
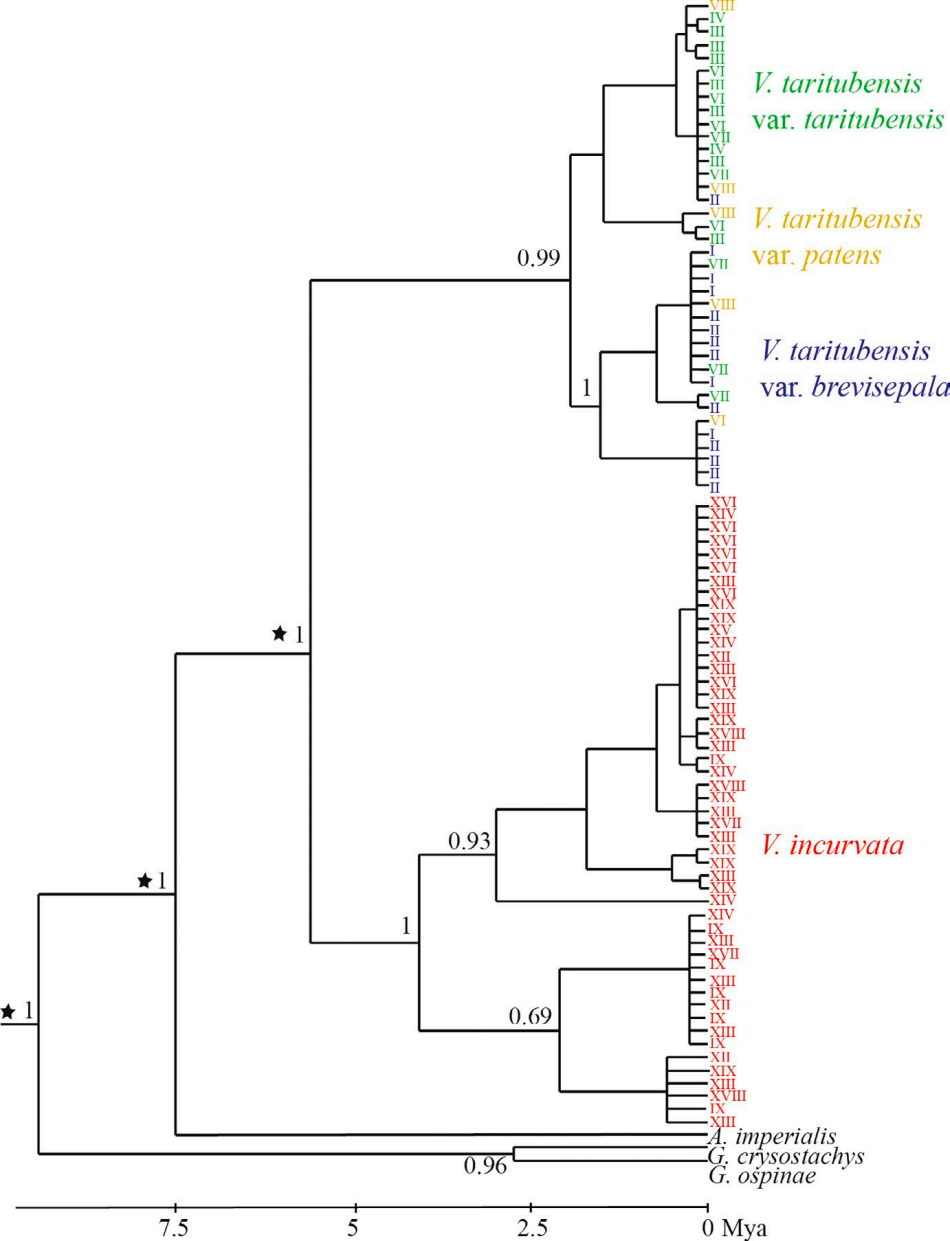
Maximum Clade Credibility tree from BEAST based on *trnL‐trnF* and *matK* sequences with posterior probabilities above 0.8 shown for the main clades. Two major clades splitting *V. taritubensis* (green, yellow and blue) and *V. incurvata* (red) specimens are recovered. Stars represent calibration points. For localities number see Table S1 in Appendix S2 [Colour figure can be viewed at http://wileyonlinelibrary.com]

The Bayesian consensus tree showed that *V. taritubensis* and *V. incurvata* likely diverged around 5 million years ago (Mya) during Late Quaternary (Pliocene), and *V. taritubensis* varieties more recently (Figure 5). Two well‐supported clades comprised both *V. taritubensis* (PP = 0.99) and *V. incurvata* (PP = 1) specimens. *Vriesea taritubensis* individuals tend to cluster in two major subclades splitting *V. taritubensis* var. *taritubensis* and *V. taritubensis* var. *brevisepala*, with few exceptions. While individuals *V. taritubensis* var. *patens* are spread along the two subclades. However, the clade comprising most individuals from *V. taritubensis* var. *taritubensis* lacks strong posterior probability support (Figure 5).

### 3.3 Predictors of morphological variation

Temperature seasonality and genetics were the main drivers of floral bract shape and together explained a large proportion of the trait variance (66%). Annual precipitation, temperature seasonality and genetics were recovered as drivers of leaf size changes and our best fit model for leaf size explained 28% of overall variation. Only a small portion of the variation was predicted by bract size and leaf shape models (6% and 9%, respectively) and both traits correlated with genetics and altitude (Table 3). For complete information on best models selected see Table S4, Appendix S2.

**Table 3:** Results of the best models selected based on AIC to explain morphological trait variation in *Vriesea incurvata* complex along the Atlantic Forest

Full initial model: response variable ~ tempseason + annualprec + alt + pco1gen + pco2gen				
Selected model: bractshape ~ tempseason + pco2gen				*R* ^2^adj = 0.66
	Estimate	SE	Pr(>t)	Sample size = 122
tempseason	0.5004	0.1243	0.0001***	
pco2gen	0.3158	0.0534	<0.0001***	
Selected model: bractsize ~ annualprec + alt + pco2gen				*R* ^2^adj = 0.06
	Estimate	SE	Pr(>t)	Sample size = 122
annualprec	−0.2290	0.1325	0.0864	
alt	−0.2409	0.0925	0.0104 *	
pco2gen	−0.3147	0.1290	0.0162 *	
Selected model: leafshape ~ alt + pco2gen				*R* ^2^adj = 0.09
	Estimate	SE	Pr(>t)	Sample size = 109
alt	−0.2557	0.0917	0.00625 **	
pco2gen	0.2431	0.0917	0.00922 **	
Selected model: leafsize ~ tempseason + annualprec + pco1gen + pco2gen				*R* ^2^ adj = 0.28
	Estimate	SE	Pr(>t)	Sample size = 122
tempseason	−0.6515	0.1990	0.00143 **	
annualprec	−0.6104	0.1233	<0.0001***	
pco1gen	−0.3656	0.2068	0.0799	
pco2gen	−0.4916	0.1168	<0.0001***	

Note

The variance explained by the selected model for each response variable is given (*R* ^2^). The explaining factors are marked according to significance level (*0.01, **0.001, ***0). In bract shape model we included PCNM1 to control for spatial autocorrelation (not shown, see methods section). The full initial model included all explanatory factors: tempseason, temperature seasonality; annualprec, annual precipitation; alt, altitude; pco1gen, PCo 1 of genetics; pco2gen, PCo 2 of genetics.

## 4 DISCUSSION

We used an interdisciplinary approach to investigate the factors underlying morphological trait variation in bromeliads across a latitudinal gradient in the Atlantic Forest. Temperature seasonality is the main driver of floral bract shape and together with annual precipitation explains leaf size variation. The genetic structure (Figure 1b and Figure S2a) correlates with morphological trait variation and all models indicate an influence of genetic inheritance (Table 3). This finding rejects our two hypotheses as follows (a) vegetative and reproductive characters are both labile and (b) shape is not more conserved than size, and they both vary with environmental heterogeneity. Our results also reveal a north–south phylogeographical break between the Bocaina region and southern portion of Atlantic Forest (Figures 1 and 5), from where *V. taritubensis* and *V. incurvata* likely diverged around 5 Mya. Divergence was likely a consequence of tectonic events that continued shaping the complex topography of the Serra do Mar at that time, resulting in marked habitat heterogeneity (Brunes, Sequeira, Haddad, & Alexandrino, 2010; Ribeiro, 2006).

### 4.1 Climatic drivers of morphological traits

We found clear trends in shape and size that are congruent with climatic variation along the Atlantic Forest latitudinal gradient (Figure 1). Floral bracts are wide‐elliptic and leaf sizes larger (wider blades and sheaths) in localities at highly seasonal sites with lower precipitation levels (*V. incurvata* distribution) in the southern, subtropical portion of the Atlantic Forest (Figures 1, 2, 3 and 4). This phenotype relates to bracts and rosettes with greater capacity to intercept and accumulate water, ensuring survival in dryer periods. In contrast, our data show lanceolate bracts and narrower leaf blades and sheaths in localities at the Bocaina region (*V. taritubensis* var. *taritubensis* and *V. taritubensis* var. *patens* distribution), where climate is hot most of the year and annual precipitation reaches the highest levels in the studied area (up to 2,587 mm). The high rainfall availability throughout the year selects for narrow bracts and leaf blades, behaving like gutters for water drainage. An intermediate morphology of elliptic bracts is found at the northernmost distribution (*V. taritubensis* var. *brevisepala* distribution), where annual precipitation is moderate (ranging from 1,336 to 1,740 mm) and there is little seasonal temperature variation (Figures 1 and 2).

The size and shape of leaves have been widely associated with temperature and moisture variables (Nicotra et al., 2011; Royer, Wilf, Janesko, Kowalski, & Dilcher, 2005; Wright et al., 2017). A global assessment of climatic drivers of leaf size demonstrated no effective thermal constraint acting in warm and ever‐wet tropical climates, as sufficient water is commonly available for transpirational cooling and plants are not exposed to frost damage (Wright et al., 2017). At a small scale and focusing on a clade of understorey epiphytes in a humid mountain tropical forest, we still detect climate influencing leaf size changes (Table 3). Besides considering leaf area itself, bromeliad tanks store water that is then available for a longer time period than a single rainfall event. Being large leaved at seasonal sites with lower mean annual precipitation is advantageous if this size translates into wider blades and sheaths to form a robust tank to retain water. Our results contrast to the pattern of a decrease in size at sites with lower rainfall and hotter temperatures with increased irradiance (Peppe et al., 2011; Ribeiro et al., 2016; Wright et al., 2017), but is consistent if interpreted in a more comprehensive way regarding plant architecture. Furthermore, the rainwater drainage capacity of the narrow‐leaved plants generally found in humid regions can prevent leaf surface water retention, which can affect gas exchange and inhibit photosynthesis (Benzing, 2000; Martin & Siedow, 1981). Bromeliads show a large variety of leaf shape and size and tank forms (Benzing, 2000). We hypothesize the pattern we found here to occur in another epiphytic tank‐forming bromeliads which are widely distributed across Atlantic Forest.

We show bract shape changes follow similar trends as leaf size in being strongly correlated with temperature seasonality (Table 3; Figures 1 and 2). Contrary to our findings, Pélabon et al. (2011) found leaf size to be more sensitive to environment than floral bract size. The bracts of *V. incurvata* complex are leaf‐like, boat‐shaped and distichally arranged along the inflorescence axis. Such display is similar to the water impound tank of most bromeliads and likely controls flower humidity, reflecting the similar pattern of variation found for the leaves.

Floral bract size and leaf shape variation were poorly explained by our models (Table 3). Both traits show high local and intraspecific variation that could be influenced by other abiotic local conditions and/or biotic interaction, or still by stochastic factors not accounted for in this study. Leaf shape is shown to be driven by both evolutionary and environmental forces (Chitwood & Sinha, 2016). We identified some influence of both genetic structure and climate driving leaf shape, despite the diffuse pattern recovered among taxa and along the latitudinal gradient in the forest (Table 3, Figure 4). Finally, most morphometric studies assessing leaf shape variation have been focused on individual taxa and eudicotyledons net‐veined leaves with distinct petioles (Royer, Meyerson, Robertson, & Adams, 2009). Our study is one of the first to present an integrative and ecological assessment of shape of leaves and floral bracts in bromeliads and, as far as we know, in monocotyledons.

### 4.2 Genetic structure and diversity reflect morphological and taxon divergence

Our models show a genetic component for all the traits tested (Table 3) and genetic structure corresponds with morphological trait variation in the *V. incurvata* complex. We found strong genetic structure among taxa with the exception of *V. taritubensis* var. *taritubensis* and *V. taritubensis* var. *patens,* which show low genetic differentiation (Table 2, Figures 1 and 5). *Vriesea incurvata* and *V. taritubensis* started to diverge in the Pliocene (5 Mya; Figure 5) and probably experienced a long period of genetic isolation in consequence of geographical and climatic barriers, and/or influenced by low or absent gene flow.

On the other hand, the *V. incurvata* and *V. taritubensis* varieties showed moderate to high intraspecific genetic diversity and intraspecific gene flow (Table 1; Tables S1 and S3, Figure S2, Appendix S2), as has been observed for other bromeliad species (Goetze, Zanella, Palma‐Silva, Büttow, & Bered, 2017; Palma‐Silva et al., 2009; Zanella et al., 2016). The low genetic variation among localities within taxa suggests that intra‐taxon gene flow has been maintained (Figure S2, Appendix S2). The *V. incurvata* complex is pollinated by hummingbirds such as *Phaethornis eurynome*, generalist and non‐territorial long‐distance dispersers (Machado & Semir, 2006). Seeds are wind dispersed, which may also facilitate the maintenance of gene flow and localities connectivity.

We show floral bract shape and chloroplast divergence support different taxa, except for *V. taritubensis* var. *taritubensis* and *V. taritubensis* var. *patens* that share haplotypes and the same floral bract shape (Figure 1). The two species *V. incurvata* and *V. taritubensis* are clearly distinct from each other and the varieties of *V. taritubensis* are not monophyletic (Figure 5). Our results partly corroborate the recent taxonomic circumscription proposed by Neves et al. (2018) using traditional morphometrics.

The new variety *V. taritubensis* var. *patens* proposed by these authors was described based on size and orientation of the inflorescence. We found three exclusive haplotypes for *V. taritubensis* var. *patens* and a difference in size separating it from *V. incurvata* (Figures 1b and 3a). Our PCoA results based on pairwise *F*_ST_ distance across all localities also showed a separation of *V. taritubensis* var. *patens* (locality VIII) along PCo 2 (Figure S2a, Appendix 1). Despite this lack of evidence we find here for *V. taritubensis* var. *patens*, we suggest taxonomic decisions be made using more robust molecular data.

### 4.3 Phylogenetic and biogeographical patterns in the *Vriesea incurvata* complex

We identify a north–south phylogeographical break between the Bocaina region and southern Atlantic Forest (Figures 1 and 5). The divergence between *V. incurvata* and *V. taritubensis* started around 5 Mya, followed by the split of the *V. taritubensis* varieties around 2.5 Mya, during the Pliocene–Early Pleistocene (Figure 5). This temporal framework is in accordance with that estimated by Kessous et al. (2019) for *Vriesea* species diversification (starting ca. 3 Mya and increasing during Pleistocene) and also coincides with the divergence of other widely distributed Atlantic Forest lineages such as reptiles and amphibians (Brunes et al., 2010; Fitzpatrick, Brasileiro, Haddad, & Zamudio, 2009; Grazziotin, Monzel, Echeverrigaray, & Bonatto, 2006).

The topography of the Brazilian east coast, specifically the Serra do Mar mountains, was formed during the Cretaceous and has been continuously modified by tectonic activity and erosional processes until the Pliocene (ca. 5–3 Mya) (Almeida, 1976). These geological processes together with the subsequent Pleistocene climatic oscillations likely facilitated species divergence (e.g. Turchetto‐Zolet et al., 2013).

The central region of the Atlantic Forest was recognized as a “hotspot within a hotspot” based on palaeoclimatic data (Carnaval, Hickerson, Haddad, Rodrigues, & Moritz, 2009). The phylogeographical break that split *V. incurvata* and *V. taritubensis* occurs in this region (between 15° and 23°S) and was reported for other *Vriesea* species, although at different locations (Palma‐Silva et al., 2009; Zanella, 2013). We suggest the break to be the result of increased topographic discontinuity of the Serra do Mar mountains, which led to geographical isolation. Furthermore, the central Atlantic Forest is a convergence zone of numerous distinct bromeliad lineages and is highly species rich (Fontoura, Scudeller, & Costa, 2012), consistent with the higher biodiversity levels found in the central portion (Carnaval et al., 2009).

Pollen data from Holocene vegetation support the existence of historical refugia in the Serra da Bocaina and Serra dos Órgãos (Behling, Dupont, Safford, & Wefer, 2007; Behling & Safford, 2010), where three varieties of *V. taritubensis* are distributed. Niche models for *V. incurvata* showed climatic stability of the southern portion of the Atlantic Forest during Last Glacial Maximum (Aguiar‐Melo et al., 2019). Lineages in the *V. incurvata* complex have likely survived in stable areas during the more recent climatic oscillations of Quaternary and from there colonized its current range.

## 5 CONCLUSIONS

Our findings identify the role of climate, genetics and topography in shaping morphological traits in the Atlantic Forest. We provide a broad‐scale quantification of trait variability at both inter‐ and intraspecific levels. Importantly, we show that reproductive (floral bracts) and vegetative (leaves) traits respond in similar ways to abiotic factors, contradicting previous suggestions. Temperature seasonality and rainfall regime are associated with the morphological diversification of these bromeliads as they drive traits variation that are diagnostic for taxa and have an ecological role. Morphological variation and genetic structure patterns coincided with recurrent biogeographical breaks in space (central region) and time (Late Pliocene) with other widely distributed Atlantic Forest organisms. We shed new light on the species delimitation of one of many species complexes of morphologically similar taxa within *Vriesea*. Because the Atlantic Forest is highly threatened and suffers continued habitat destruction, studies like this help to unveil combined evolutionary and ecological dynamics generating species diversification, before much biodiversity is lost.

## BIOSKETCH

**Beatriz Neves** is interested in the evolution and diversification of bromeliads in the Neotropics. The research team studies systematics of Bromeliaceae, population genetics of Neotropical plants, and the past, present and future of biodiversity.

Author contributions: B.N. collected the morphological data, and C.M.Z. the genetic data. A.F.C., B.N., C.D.B. and A.A. conceived the ideas. B.N. and C.M.Z. performed the analyses. I.M.K. built the phylogeny. All authors contributed to interpreting and discussing results and writing the manuscript.

## Data Availability

The data that support the findings of this study will be openly available in the DRYAD Digital Repository. Neves, B., Zanella, C. M., Kessous, I. M., Uribbe, F. P., Salgueiro, F., Bered, F., Antonelli, A., Bacon, C. D., & Costa, A. F. (2019). Seasonality drives bromeliad leaf and floral bract variation across a latitudinal gradient in the Atlantic Forest. (https://doi.org/10.5061/dryad.sj3tx9619).
