# Supplementary figures and images for: Drivers of bromeliad leaf and floral bract variation across a latitudinal gradient in the Atlantic Forest
Source: J Biogeogr. 2019 Nov 24;47(1):261–74. doi: 10.1111/jbi.13746 (PMC7006768; doi:10.1111/jbi.13746)

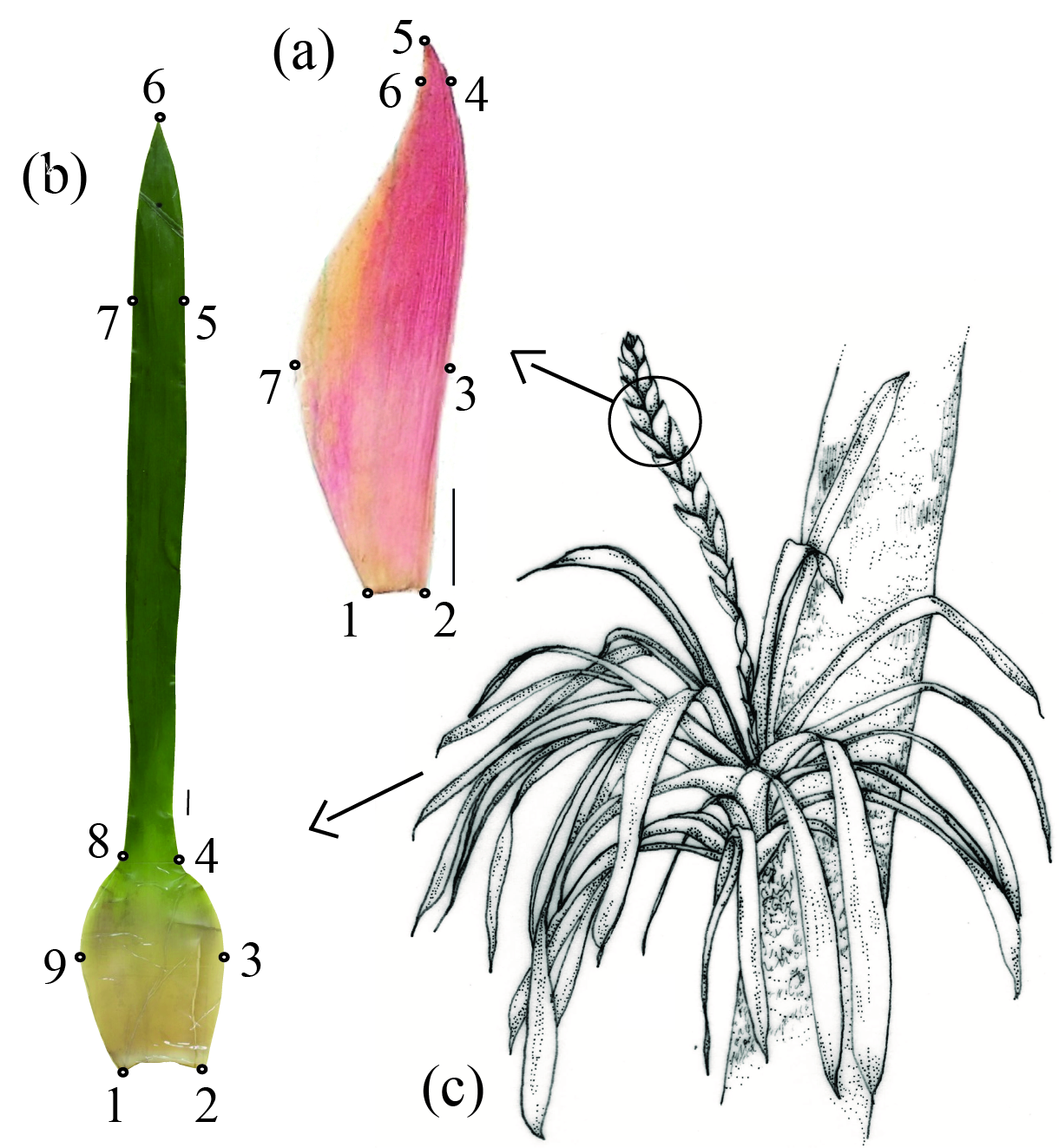

Supplement: Supplementary file 1 [file JBI-47-261-s001.tif]

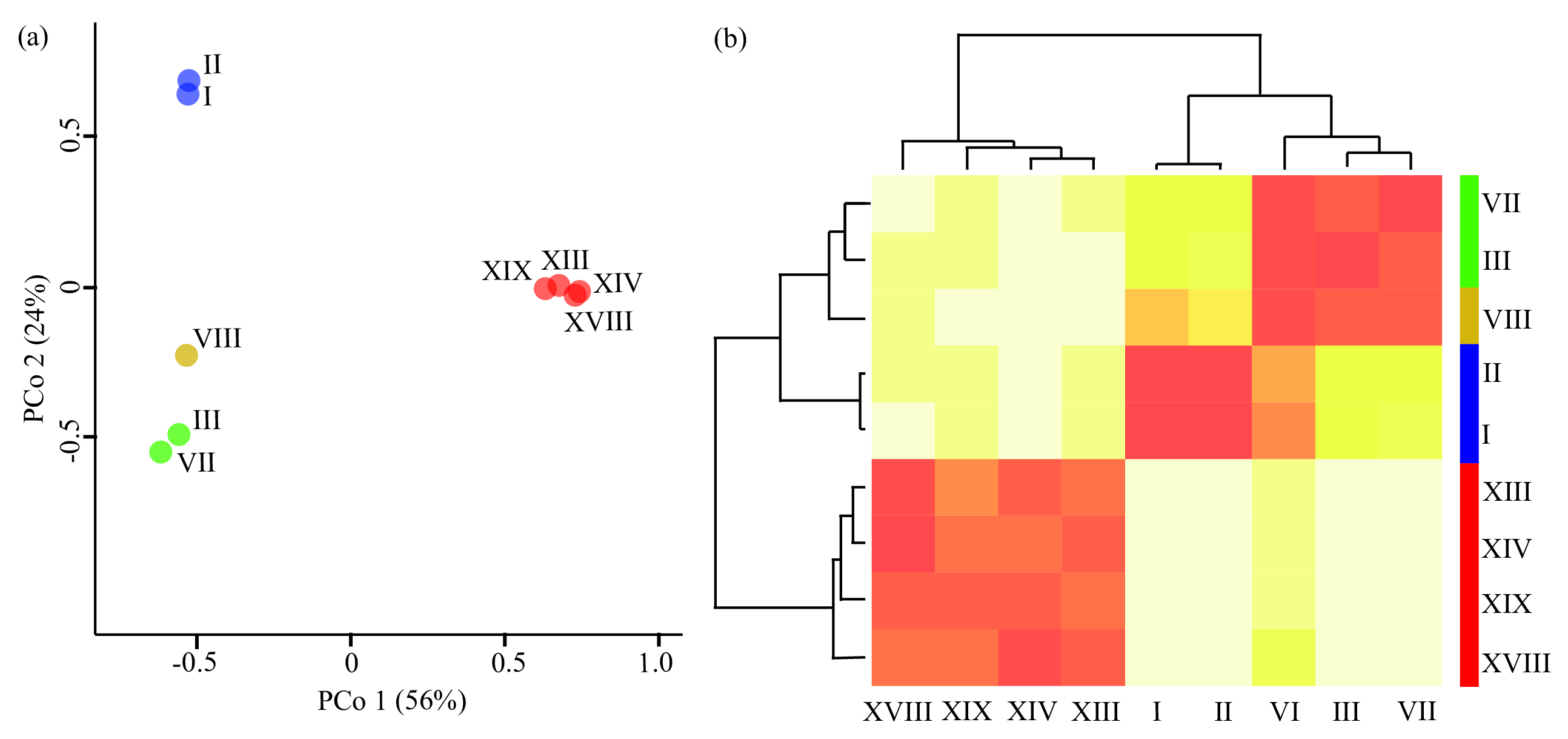

Supplement: Supplementary file 2 [file JBI-47-261-s002.tif]
