# Supplementary material for: Drivers of bromeliad leaf and floral bract variation across a latitudinal gradient in the Atlantic Forest
Source: J Biogeogr. 2019 Nov 24;47(1):261–74. doi: 10.1111/jbi.13746 (PMC7006768; doi:10.1111/jbi.13746)
Supplement: Supplementary file 3 [file JBI-47-261-s003.doc]

*Journal of Biogeography*

**Supporting information**

Drivers of bromeliad leaf and floral bract variation across a latitudinal gradient in the Atlantic Forest

Beatriz Neves, Camila M. Zanella, Igor M. Kessous, Fernando P. Uribbe, Fabiano Salgueiro, Fernanda Bered, Alexandre Antonelli, Christine D. Bacon & Andrea F. Costa

**APPENDICES**

**Appendix S1.** Description of the results of PCA performed to select the environmental variables to be included in the models.

From the PCA analysis of the 19 bioclimatic variables and altitude, the first three principal components explained 96% of the total variation. The loadings of PC1 and PC2 (accounting for 53.6% and 23.1% of variance, respectively) were highly correlated with temperature seasonality (BIO4) and annual precipitation (BIO12). 20% of the variance retained by PC3 was almost completely explained by altitude (Table S2, Appendix S2). Annual precipitation (the sum of all total monthly precipitation) ranged from 1,337 to 2,587 mm, while temperature seasonality (the amount of temperature variation over a year based on the standard deviation of monthly temperature averages, with standard deviation *100) ranged from 2,111 to 3,269.

**Appendix S2.** Supplementary tables and figures.

**TABLE S1** Taxa, localities, geographic coordinates, sampling number for morphology (bract and leaf) and for molecular analysis, and haplotypes found in 20 populations of *Vriesea taritubensis* var. *brevisepala, V. taritubensis* var. *taritubensis, V. taritubensis var. patens* and *V. incurvata*.

|  |  | |  |  | N Sampling | |  |
| --- | --- | --- | --- | --- | --- | --- | --- |
| Taxa | Locality # | Long | Lat | | Morpho Bract/Leaf | Gene | Haplotypes |
| *V. taritubensis* var. *brevisepala* | I- Rio de Janeiro (RJ), Magé, Coruja-italianos | -43.0330 | -22.5269 | | 18/14 | 06 | H24, H25 |
|  | II- Rio de Janeiro (RJ), Cachoeiras de Macacu | -42.8044 | -22.5380 | | 17/11 | 10 | H24, H26, H27 |
| *V. taritubensis* var. *taritubensis* | III- Rio de Janeiro (RJ), Paraty, Fazenda São Gonçalo | -44.6130 | -23.0258 | | 13/13 | 07 | H14, H20, H21, H22, H23 |
|  | IV- Rio de Janeiro (RJ), Paraty, Camburi | -44.7131 | -23.2178 | | - | 02 | H14, H17 |
|  | V- Rio de Janeiro (RJ), Paraty, Corisco | -44.7894 | -23.3086 | | 19/14 | - |  |
|  | VI- São Paulo, (SP), Ubatuba, Poço dos Amores | -45.0833 | -23.4333 | | - | 04 | H17, H18, H19 |
|  | VII- São Paulo (SP), Ubatuba, Cachoeira do Ipiranguinha | -45.1287 | -23.4279 | | 12/11 | 05 | H11, H14 |
| *V. taritubensis* var. *patens* | VIII- São Paulo (SP), São Luís do Paraitinga | -45.1470 | -23.3376 | | 18/14 | 06 | H11, H12, H13, H14, H15, H16 |
| *V. incurvata* | IX- São Paulo (SP), São Paulo, Rodoanel* | -46.7833 | -23.4500 | | - | 07 | H1, H2, H10 |
|  | X- São Paulo (SP), Salesópolis | -45.8877 | -23.6562 | | 19/13 | - |  |
|  | XI- São Paulo (SP), Ribeirão Grande | -48.4052 | -24.2717 | | 13/12 | - |  |
|  | XII- São Paulo (SP), Cananeia* | -47.8108 | -24.6537 | | - | 03 | H1, H2 |
|  | XIII- Paraná (PR), Morretes, Serra da Graciosa* | -48.8858 | -25.3473 | | 15/14 | 12 | H1, H2, H4, H8, H9 |
|  | XIV- Paraná (PR), Matinhos* | -48.5285 | -25.7889 | | 08/08 | 05 | H1, H2 |
|  | XV- Santa Catarina (SC), Joinville | -49.0189 | -26.0965 | | 14/12 | - |  |
|  | XVI- Santa Catarina (SC), Corupá* | -49.3441 | -26.4020 | | - | 07 | H1, H6, H7 |
|  | XVII- Santa Catarina, (SC), Antônio Carlos* | -48.8682 | -27.4567 | | - | 02 | H2, H5 |
|  | XVIII- Santa Catarina (SC), Florianópolis* | -48.5073 | -27.5246 | | 13/13 | 03 | H1, H2 |
|  | XIX- Rio Grande do Sul (RS), Maquiné, Cachoeira do Garapiá* | -50.2398 | -29.5048 | | 13/14 | 09 | H1, H2, H3, H4 |
|  | XX- Rio Grande do Sul (RS), Santo Antônio da Patrulha | -50.2717 | -29.7387 | | 16/13 | - |  |

*Molecular data generated by Zanella et al. (2016). Hybrids between *V. incurvata* and *V. carinata* were found in localities XIII and XIV and were excluded from our analyses.

**TABLE S2** PCA loadings of the 19 climate biovariables extracted from Worldclim database, and altitude. The correlated and less explicative were excluded from the main analyses. In bold the selected ones.

| Environmental Variables | PC1 | PC2 | PC3 |
| --- | --- | --- | --- |
| BIO1Annual Mean Temperature | 0.0245 | -0.02194 | 0.03215 |
| BIO2 Mean Diurnal Range (Mean of monthly (max temp - min temp)) | 0.004775 | -0.00813 | -0.01933 |
| BIO3 Isothermality (BIO2/BIO7) (* 100) | 0.004955 | -0.00632 | -0.00886 |
| BIO4 **Temperature Seasonality (standard deviation *100)** | **-0.6015** | **0.5489** | **0.4874** |
| BIO5 Max Temperature of Warmest Month | 0.01844 | -0.02052 | 0.03628 |
| BIO6 Min Temperature of Coldest Month | 0.02546 | -0.02502 | 0.04414 |
| BIO7 Temperature Annual Range (BIO5-BIO6) | -0.00702 | 0.0045 | -0.00785 |
| BIO8 Mean Temperature of Wettest Quarter | 0.03037 | -0.01905 | 0.02028 |
| BIO9 Mean Temperature of Driest Quarter | 0.02174 | -0.02623 | 0.04241 |
| BIO10 Mean Temperature of Warmest Quarter | 0.01791 | -0.01561 | 0.04017 |
| BIO11 Mean Temperature of Coldest Quarter | 0.03201 | -0.02908 | 0.02753 |
| BIO12 **Annual Precipitation** | **0.6273** | **0.5904** | 0.1928 |
| BIO13 Precipitation of Wettest Month | 0.09663 | 0.03831 | -0.01483 |
| BIO14 Precipitation of Driest Month | -0.00071 | 0.0455 | 0.04047 |
| BIO15 Precipitation Seasonality (Coefficient of Variation) | 0.01374 | -0.01905 | -0.02203 |
| BIO16 Precipitation of Wettest Quarter | 0.307 | 0.1263 | -0.03773 |
| BIO17 Precipitation of Driest Quarter | -0.0096 | 0.1598 | 0.1212 |
| BIO18 Precipitation of Warmest Quarter | 0.2886 | 0.1449 | -0.01664 |
| BIO19 Precipitation of Coldest Quarter | -0.02243 | 0.1708 | 0.1339 |
| **Altitude** | -0.2292 | **0.5008** | **-0.8242** |

**TABLE S3** AMOVA results from plastid DNA data (*matK* + *trnL-trnF*) showing the higher amount of variation among taxa.

| Source of variation | Percentage of variation | *F* statistics | *P*-value |
| --- | --- | --- | --- |
| Among species | 79.66 | *F*_ct_ 0.796 | 0.0000 |
| Among localities within species | 2.6 | *F*_st_ 0.822 | 0.0000 |
| Within localities | 17.74 | *F*_sc_ 0.127 | 0.0006 |

**TABLE S4** Results of the best models selected for each response variable. We give the beta estimate for each predictor, df and values of AICc (AIC corrected to low sample sizes). We show only models with delta < 2 for each response variable, such models should be considered informative when choosing for the best model (see Burnham & Anderson, 2002). We interpret the top model as the best one for each response variable and give their statistics in the Table 3 of the main manuscript.

| **Bract shape** |  |  |  |  |  |  |  |  |  |  |
| --- | --- | --- | --- | --- | --- | --- | --- | --- | --- | --- |
| Intercept | Temperature seasonality | Annual precipitation | Altitude | PCo 1 genetics | PCo 2 genetics | PCNM1 | df | AICc | delta | weight |
| 2.79E-16 | 0.5004 | - | - | - | 0.3158 | 0.3058 | 5 | 220.5 | 0 | 0.2 |
| 8.69E-17 | 0.4978 | - | - | -0.304 | 0.295 | - | 5 | 221 | 0.51 | 0.155 |
| 1.27E-16 | - | - | -0.06297 | -0.2615 | 0.2978 | 0.5411 | 6 | 221.8 | 1.37 | 0.101 |
| 2.95E-16 | 0.5447 | - | -0.04578 | - | 0.315 | 0.2601 | 6 | 222 | 1.58 | 0.091 |
| **Bract size** |  |  |  |  |  |  |  |  |  |  |
| Intercept | Temperature seasonality | Annual precipitation | Altitude | PCo 1 genetics | PCo 2 genetics | df | AICc | delta | weight |  |
| 2.84E-16 | - | -0.229 | -0.2409 | - | -0.3147 | 5 | 345 | 0 | 0.162 |  |
| 3.35E-16 | - | - | -0.1922 | - | -0.1518 | 4 | 345.8 | 0.88 | 0.105 |  |
| 7.54E-16 | 0.4525 | -0.3387 | -0.317 | 0.4351 | -0.3768 | 7 | 345.9 | 0.9 | 0.104 |  |
| 3.15E-16 | 0.0652 | -0.2129 | -0.2419 | - | -0.2976 | 6 | 346.6 | 1.67 | 0.071 |  |
| 3.89E-16 | - | - | -0.1955 | - | - | 3 | 346.7 | 1.7 | 0.07 |  |
| **Leaf shape** |  |  |  |  |  |  |  |  |  |  |
| Intercept | Temperature seasonality | Annual precipitation | Altitude | PCo 1 genetics | PCo 2 genetics | df | AICc | delta | weight |  |
| -1.64E-16 | - | - | -0.2557 | - | 0.2431 | 4 | 303.4 | 0 | 0.187 |  |
| -2.08E-16 | -0.1129 | - | -0.2425 | - | 0.2381 | 5 | 304.1 | 0.64 | 0.136 |  |
| -1.20E-16 | - | - | -0.2576 | 0.09775 | 0.2427 | 5 | 304.4 | 1.02 | 0.113 |  |
| -1.81E-17 | - | -0.2426 | -0.3034 | 0.1531 | - | 5 | 305.3 | 1.84 | 0.075 |  |
| **Leaf size** |  |  |  |  |  |  |  |  |  |  |
| Intercept | Temperature seasonality | Annual precipitation | Altitude | PCo 1 genetics | PCo 2 genetics | df | AICc | delta | weight |  |
| 2.63E-16 | -0.6515 | -0.6104 | - | -0.3656 | -0.4916 | 6 | 287.6 | 0 | 0.319 |  |
| -1.61E-16 | -0.3225 | -0.747 | -0.1431 | - | -0.5667 | 6 | 287.8 | 0.23 | 0.284 |  |
| -1.53E-16 | -0.3307 | -0.6925 | - | - | -0.5401 | 5 | 288.6 | 0.99 | 0.194 |  |
| 1.37E-16 | -0.5528 | -0.6706 | -0.0956 | -0.2593 | -0.5234 | 7 | 288.8 | 1.19 | 0.176 |  |

Note: Burnham, K. P. & Anderson, D. R. (2002). Model selection and multimodel inference: a practical information-theoretic approach. New York: Springer-Verlag.


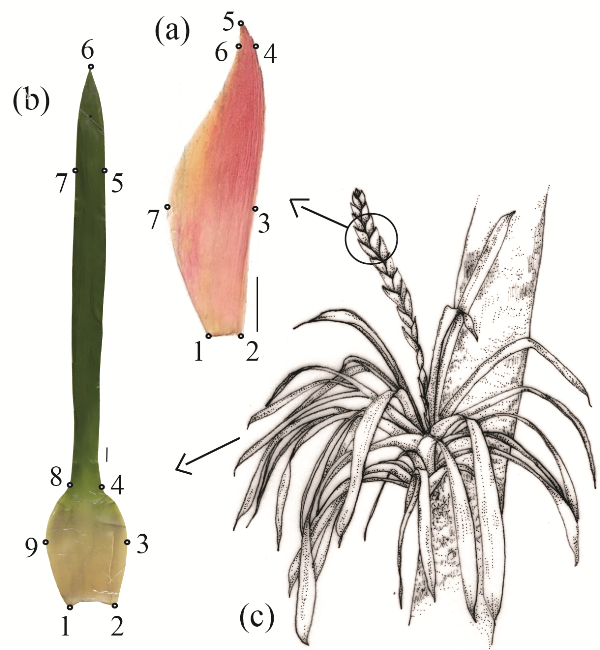


**FIGURE S1** Location of landmarks on floral bract profile and leaf. (a) At the base (1, 2), widest point of the margin and its dorsal opposite (7, 3), midpoint of margin contraction and its dorsal opposite (6, 4), and apex of the floral bract profile (5). (b) At the sheath base (1, 2), sheath widest points (3, 9), transition sheath/ blade (4, 8), level ¾ of blade (5, 7), and apex of leaf (6). (c) Illustration of *Vriesea taritubensis* var. *taritubensis* by Mônica Claro.

**
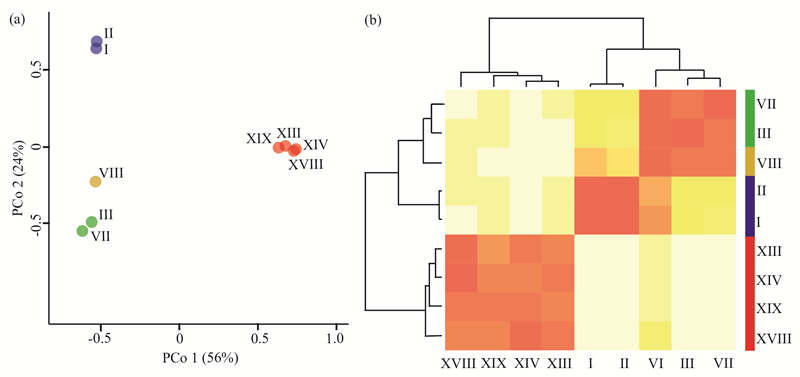
**

**FIGURE S2** Genetic distance across localities calculated from pairwise *F*_ST_ denoting the three distinct taxa: I-II *V. taritubensis* var. *brevisepala* in blue, III and VII *V. taritubensis* var. *taritubensis* in green, VIII *V. taritubensis* var. *patens* in yellow, XIII, XIV, XVIII and XIX *V. incurvata* in red. (a) A Principal Coordinate Analysis plot showing axes percentage of variation and (b) a Cluster heatmap with dark red indicating the lowest and light yellow indicating the highest genetic distance.
